# Lung epithelial stem cells and their niches: Fgf10 takes center stage

**DOI:** 10.1186/1755-1536-7-8

**Published:** 2014-05-08

**Authors:** Thomas Volckaert, Stijn De Langhe

**Affiliations:** 1 Department of Pediatrics, Division of Cell Biology, National Jewish Health, 1400 Jackson St, Denver, CO 80206, USA; 2 The Inflammation Research Center, Unit of Molecular Signal Transduction in Inflammation, VIB, Technologiepark 927, 9052 Ghent, Belgium; 3 Department of Biomedical Molecular Biology, Ghent University, Technologiepark 927, 9052 Ghent, Belgium; 4 Department of Cellular and Developmental Biology, School of Medicine, University of Colorado Denver, 12605 E 16th Avenue, Aurora CO 80045, USA

## Abstract

Throughout life adult animals crucially depend on stem cell populations to maintain and repair their tissues to ensure life-long organ function. Stem cells are characterized by their capacity to extensively self-renew and give rise to one or more differentiated cell types. These powerful stem cell properties are key to meet the changing demand for tissue replacement during normal lung homeostasis and regeneration after lung injury. Great strides have been made over the last few years to identify and characterize lung epithelial stem cells as well as their lineage relationships. Unfortunately, knowledge on what regulates the behavior and fate specification of lung epithelial stem cells is still limited, but involves communication with their microenvironment or niche, a local tissue environment that hosts and influences the behaviors or characteristics of stem cells and that comprises other cell types and extracellular matrix. As such, an intimate and dynamic epithelial-mesenchymal cross-talk, which is also essential during lung development, is required for normal homeostasis and to mount an appropriate regenerative response after lung injury. Fibroblast growth factor 10 (Fgf10) signaling in particular seems to be a well-conserved signaling pathway governing epithelial-mesenchymal interactions during lung development as well as between different adult lung epithelial stem cells and their niches. On the other hand, disruption of these reciprocal interactions leads to a dysfunctional epithelial stem cell-niche unit, which may culminate in chronic lung diseases such as chronic obstructive pulmonary disease (COPD), chronic asthma and idiopathic pulmonary fibrosis (IPF).

## Review

### Region-specific stem cells maintain and repair the adult lung epithelium

**Figure 1:**
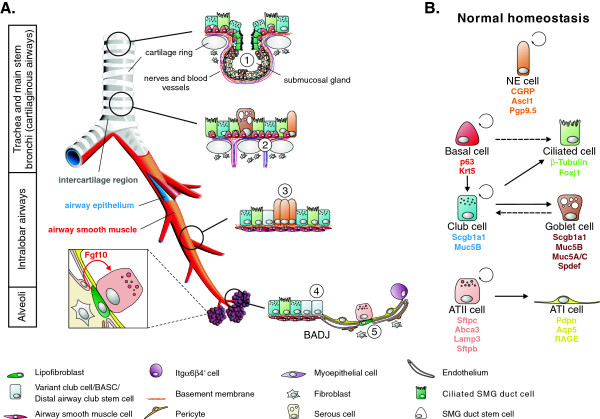
**The composition of the adult mouse lung epithelium during normal homeostasis. (A)** The mouse lung is organized into three anatomical regions. The cartilaginous airways (trachea and main stem bronchi) are lined by a pseudostratified epithelium consisting of secretory (club and goblet), ciliated, basal and a few scattered neuroendocrine (NE) cells. Submucosal glands (SMGs) are located between cartilage rings of the proximal trachea and contain a stem cell population in their ducts (1). Label-retaining basal stem cells are often found in the intercartilage regions (2). The intralobar airway epithelium contains club, ciliated and clusters of NE cells called NE bodies (NEBs), which are often found at branching points. Naphthalene-resistant (variant) club cells are located adjacent to the NEBs (3) and at the bronchioalveolar duct junctions (BADJs) (4), and are presumed to be important for epithelial regeneration. The latter most likely represents a heterogeneous population containing bronchioalveolar stem cells (BASCs) and distal airway club stem cells (DASCs), which are activated after injury. The alveolar epithelium consists mainly of alveolar type (AT) I and ATII cells. The latter is a long-term self-renewing stem cell population also capable of giving rise to ATI cells. Lipofibroblasts in the lung interstitium express *Fgf10* and are found juxtaposed to ATII stem cells (5). They are therefore an ideal candidate as a niche that controls the behavior of ATII cells during normal homeostasis and after injury. In addition, the alveoli harbor an alveolar progenitor cell enriched for α6β4 integrins. **(B)** Lineage relationships of lung epithelial stem cells and their progeny during normal homeostasis. The lung epithelium is maintained by three main stem cell populations: basal cells (cartilaginous airways), club cells (cartilaginous airways and bronchioles) and ATII cells (alveoli). Dashed arrows represent lineage relationships, which are likely to occur but have not yet been definitively established. For details see main text.

The adult lung epithelium is replaced over time, albeit very infrequently in comparison to organs exhibiting constant cellular turnover such as the skin and intestine. However, after injury, the lung harbors a remarkable capacity to regenerate and restore its function. This is dramatically illustrated after unilateral pneumectomy, which induces an expansion of stem cell populations and compensatory growth of the remaining lung to re-establish respiratory capacity [1]. The composition of the lung epithelium varies along a proximal-distal axis (Figure 1A), which is reflected in the diverse physiological functions of the lung. In the mouse, the pseudostratified epithelium of the trachea and main stem bronchi consists of ciliated cells, club (also known as Clara) cells, a few mucus/goblet cells, and relatively undifferentiated basal cells, which express the transcription factor transformation-related protein 63 (Trp63 or p63), cytokeratin (Krt) 5 and/or Krt14. In the smaller intralobar bronchioles, the pseudostratified epithelium now transitions into a simple single columnar to cuboidal epithelial layer devoid of basal cells and containing mostly club and ciliated cells interspersed with single or clustered neuroendocrine (NE) cells termed NE bodies (NEBs), which are most frequently located at airway bifurcations. Of note, the basal cell-containing pseudostratified epithelium in human lungs extends to the distal bronchioles [2]. In the most distal regions of the lung, approximately 90% of the alveolar epithelium is composed of flattened alveolar type (AT) I cells, which are in close apposition to the capillary endothelium, allowing for rapid and efficient gas exchange, and cuboidal ATII cells that express surfactant. It is now becoming clear that these different epithelial regions in the lung are maintained and repaired by distinct stem cell populations.

### Maintaining lung epithelium during normal homeostasis

Lineage tracing experiments during normal homeostasis have identified three main stem cell populations responsible for maintaining the lung epithelium: basal cells, club cells and ATII cells. Their lineage relationships are depicted in Figure 1B. Basal cells in the proximal airways are a bona fide stem cell population that gives rise to club and ciliated cells [3-6]. Club cells are also able to self-renew and give rise to ciliated cells and therefore meet the stem cell criteria as well. They are the predominant cell population responsible for maintaining the bronchiolar epithelium. In the trachea however, their contribution to epithelial self-renewal seems to be minimal, and as a population, they are replaced over time by new club cells derived from basal cells [3,7]. NE cells self-renew but under normal homeostatic conditions do not give rise to other epithelial cell lineages [8]. The alveolar epithelium is maintained by ATII stem cells, which can self-renew and can give rise to ATI cells [9,10].

### Stem cell populations contributing to epithelial regeneration after lung injury

**Figure 2:**
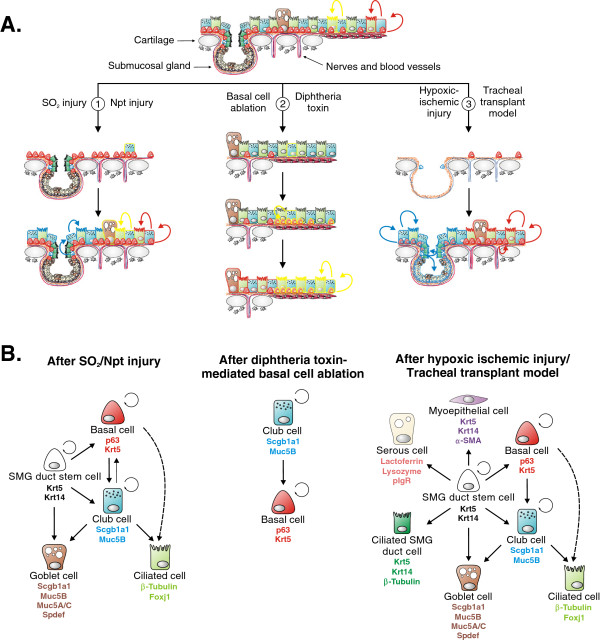
**Stem cell populations contributing to regeneration of the proximal airway epithelium. (A)** Widely used tracheal injury models such as the SO_2_, the naphthalene (Npt), and the tracheal transplant models, are used to study the contribution of stem cell populations to epithelial regeneration. SO_2_ and naphthalene injury destroy most luminal cells (1). Although surviving club cells can contribute to epithelial regeneration in the trachea following injury, the majority of newly generated club and ciliated cells arise from activated basal stem cells. A basal cell-like stem cell population residing in submucosal gland (SMG) ducts can also contribute to the regenerative process under these conditions, although they are probably employed to a larger extent after more severe injury. Although basal stem cells are presumed to be at the apex of stem cell hierarchy, club cells have been shown to be able to dedifferentiate and give rise to basal cells after diphtheria toxin-mediated depletion of the basal cell population (2). Club cell-derived basal cells can then give rise to club and ciliated cells during normal homeostasis. A more drastic epithelial injury caused by the loss of blood supply is obtained by the tracheal transplant model, which destroys nearly all epithelial cells except for a few injury resistant basal cells and SMG duct stem cells (3). After blood supply is reestablished, these surviving stem cells can then restore the tracheal surface and SMG epithelium. Colored cell outlines represent lineage trace markers. **(B)** Lineage relationships of lung epithelial stem cells and their differentiated progeny during regeneration of the tracheal epithelium after SO_2_/naphthalene injury (left), diphtheria toxin-mediated basal cell depletion (middle) and hypoxic ischemic injury using the tracheal transplant model (right). Dashed arrows represent lineage relationships, which are likely to occur but have not yet been definitively established. For details see main text.

The lung is directly exposed to the outside environment and must therefore be able to respond quickly and effectively to inhaled particles, pathogens and harmful gases. The conducting airway epithelium is therefore designed to be a crucial primary defense mechanism by mediating mucociliary clearance and forming a protective physical barrier, which maintains its structural integrity and is vital for normal lung function. By exposing mouse lungs to different types of injury it is thought that different stem cell populations are engaged, depending on the location and extent of injury. In the trachea, SO_2_ or naphthalene injury kills most luminal cell types leaving behind a few surviving club cells and an intact basal cell layer (Figure 2A). Surviving club cells proliferate and contribute to epithelial restoration, but basal cells are the most important cell type mediating tracheal regeneration under these conditions [3,7]. A Krt14^+^ basal cell-like stem cell population located in submucosal gland (SMG) ducts has been shown to drive epithelial regeneration after severe epithelial injury using a tracheal transplant model of hypoxic ischemia, which destroys nearly all epithelial cells (Figure 2A) [11,12]. This stem cell population is still poorly characterized and its further study will require more advanced lineage tracing techniques. Together, these studies have led to the notion that basal stem cells can be placed at the top of stem cell hierarchy in the trachea, a concept which has recently been complicated by the finding that diphtheria toxin-mediated ablation of basal cells in the trachea results in the reprogramming of a subset of club stem cells into basal stem cells effectively restoring the basal stem cell population of the trachea [13] (Figure 2A,B). Moreover, very rare club cell-derived basal cells have also been observed after SO_2_-mediated tracheal injury [7]. Interestingly, p63, a master regulator required for the development of basal cells [14], induces a basal cell phenotype and squamous metaplasia when ectopically expressed in club or ATII cells [15].

**Figure 3:**
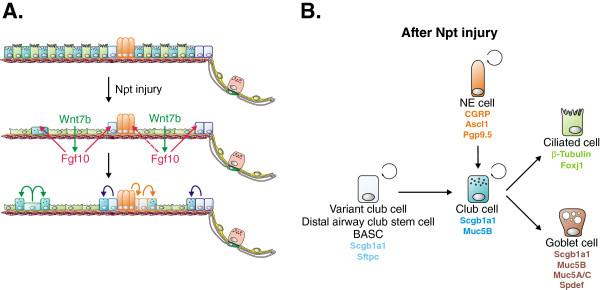
**Regeneration of the distal airway epithelium after naphthalene injury. (A)** Naphthalene (Npt) injury selectively kills all club cells in the distal conducting airways except for a few naphthalene-resistant club cells located near neuroendocrine (NE) bodies (NEBs) and at the bronchioalveolar duct junction (BADJ). Surviving ciliated cells spread out in an attempt to protect the denuded basement membrane and start to express *Wnt7b*, which then acts on airway smooth muscle cells to induce proliferation and Fgf10 secretion. Fgf10 acts back on surviving club stem cells to activate them and initiate regeneration. Thus, airway smooth muscle can be regarded as a club stem cell niche. Colored cell outlines represent lineage trace markers. **(B)** Lineage relationships of lung epithelial stem cells and their differentiated progeny during regeneration of the distal conducting airways after naphthalene injury. For details see main text. BASC, bronchioalveolar stem cell.

**Figure 4:**
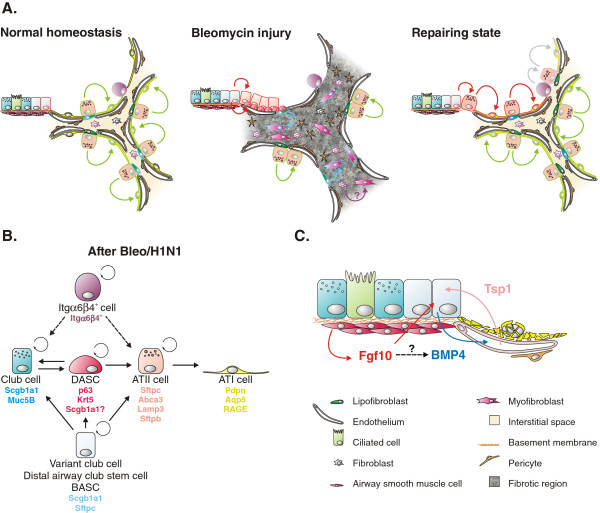
**Regeneration of alveolar epithelium after injury. (A)** Bleomycin-mediated injury results in widespread destruction of all alveolar epithelial cells. Surviving ATII cells are activated following injury, undergo proliferation and restore the alveolar epithelium. The regenerative process after bleomycin injury is associated with the progressive invasion of myofibroblasts, which form fibrotic foci featuring increased extracellular matrix deposition. Several sources for myofibroblasts have been proposed, including epithelial cells, circulating fibroblasts as well as resident (lipo)fibroblasts. Additional distal progenitor cell populations appear to contribute to the regeneration of alveolar epithelium, including Itgα6β4^+^ cells, Scgb1a1^+^ cells and distal airway stem cells (DASCs). The latter have basal cell characteristics, most likely originate from distal airway club stem cells and give rise to bronchiolar and alveolar epithelium. Colored cell outlines represent lineage trace markers. **(B)** Lineage relationships of alveolar epithelial stem cells and their differentiated progeny during regeneration of the alveolar epithelium after bleomycin injury. Dashed arrows represent lineage relationships, which are likely to occur but have not yet been definitively established. **(C)** Model highlighting molecular crosstalk between distal airway club stem cells and components of their niche, including airway smooth muscle and endothelium. For details see main text. BASC, bronchioalveolar stem cell.

A widely used model to study epithelial regeneration of the bronchioles is naphthalene injury. In the bronchioles, naphthalene selectively depletes club cells except for the few naphthalene-resistant club cells (called variant club cells in some literature) located near NEBs [16,17] and at bronchioalveolar duct junctions (BADJs) [18], which then expand and re-epithelialize the damaged airways (Figure 3A). This led to the hypothesis that this subpopulation of club cells is responsible for epithelial regeneration after injury. However, after injury, the majority of surviving club cells, regardless of their location, are capable of restoring the damaged lung epithelium [7]. Interestingly, NE cells have been shown to demonstrate some degree of plasticity and as such are capable of not only self-renewing but also to give rise to club and club cell-derived ciliated cells after airway epithelial injury [8] (Figure 3). However, elimination of pulmonary NE cells does not impair airway epithelial regeneration after naphthalene injury [8], suggesting that the contribution of NE cells to epithelial restoration is minimal. Bronchioalveolar stem cells (BASCs) are another population of naphthalene-resistant stem cells located at the BADJ and can be identified based on their coexpression of secretory (Scgb1a1) and alveolar (Sftpc) markers, [19] (Figure 1). BASCs are likely identical to the variant club stem cells located near BADJs (described above) and might also be referred to as distal airway stem cells (DASCs) [20,21]. BASCs can self-renew and give rise to both bronchiolar and alveolar cell lineages *in vitro* and *in vivo*, the latter only after catastrophic alveolar epithelial injury [19,22] (Figure 4A,B). However, lineage tracing of Scgb1a1^+^ cells during normal homeostasis or after hyperoxia injury (which selectively destroys ATI cells) did not reveal any contribution of BASCs to alveolar regeneration [7]. Using the same *Scgb1a1*^*CreER*^ mice however, it was shown that Scgb1a1^+^ club cells in the distal airway can give rise to both ATI and ATII cells following catastrophic bleomycin- or H1N1-mediated injury [20,22-25] (Figure 4A,B). Thus, it seems that the deployment of BASCs depends on the severity and type of injury. Interestingly, DASCs may dedifferentiate into basal cells after catastrophic bleomycin- or H1N1-mediated injury prior to regenerating ATI and ATII cells [20,25] (Figure 4A,B). However, it remains possible that the observed increase in basal cells is due to the expansion of a minor basal cell population in the distal airways or by migration of basal cells originating from the proximal airways. Future experiments combining both lineage tracing and live imaging during the repair response will help to address this. Bronchiolization is often observed as honeycomb regions in lungs from patients with idiopathic pulmonary fibrosis (IPF). These bronchiolized areas have been shown to contain basal cells [26], suggesting that in humans with IPF, regeneration of the fibrotic lung may also be mediated in part by distal airway club or basal stem cells.

Importantly, although BASCs/DASCs can contribute to alveolar regeneration, ATII cells are the main stem cell population in the lung respiratory epithelium involved in replenishing ATII cells after their diphtheria toxin-mediated depletion [9], re-establishing the ATI population after hyperoxic injury [7,10] and regenerating both ATI and ATII cells after bleomycin injury [9,23] (Figure 4). In addition to ATII cells, an Sftpc^−^ and integrin (Itg) α6^+^/β4^+^ alveolar epithelial stem cell population has recently been characterized, having the potential to give rise to both ATII and club cells *in vitro* and *in vivo* after injury [27,28] (Figure 4B). To what extent this cell population contributes to alveolar repair after injury is not clear and will require lineage tracing to answer this question.

### The niche regulates epithelial stem cell behavior in the lung

**Figure 5:**
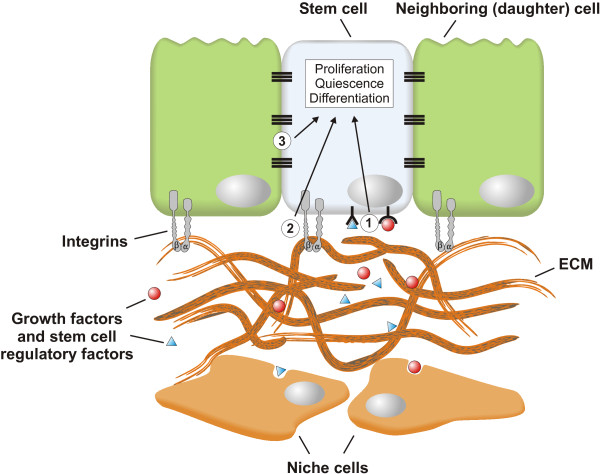
**The epithelial stem cell niche.** The behavior of epithelial stem cells is regulated by external signals, provided by the microenvironment or niche in which stem cells reside. These signals include growth factors (for example Fgf10) and other stem cell regulatory factors secreted by the niche cells, which can be a wide variety of differentiated cell types, including fibroblasts, smooth muscle cells, endothelial cells, neurons as well as neighboring stem cell progeny (1). Another important component of the stem cell niche is the extracellular matrix (ECM), which acts as a reservoir for growth factors and provides mechanical cues to stem cells, which are translated into biochemical signals through integrins via a process called mechanotransduction (2). Finally, direct cell-cell contact between stem cells and their neighboring progeny, which is mediated by adherens and tight junctions, can also provide essential feedback information to their parent stem cells (3). Integration of these different types of niche signals regulates stem cell activity and behavior such as enhancing stem cell quiescence, promoting transient proliferation or differentiation, and maintaining stem cells in an undifferentiated state.

Lung stem cells must give rise to the appropriate number of differentiated progeny to achieve homeostasis and to restore the functional organ after injury. Tissue damage can dramatically change the dimensions of an organ, and after regeneration tissue growth must halt once the original organ dimensions are restored. Therefore, checks and balances are in place to prevent unwanted stem cell responses, which could lead to pathological changes compromising tissue integrity and lung function. The behavior of virtually all stem cells, whether they are pluripotent or lineage-restricted, embryonic or adult, is controlled by the interplay between intrinsic transcriptional programs and extrinsic signals [29]. The extrinsic signals are provided by the niche, a local tissue environment that hosts and influences the behaviors or characteristics of stem cells [30] and that comprises other cell types and extracellular matrix (ECM) (Figure 5). An intimate association of stem cells with their niche is critical to ensure long-term maintenance of stem cell populations, as well as to direct stem cell differentiation into the appropriate lineages. As such, the niche can maintain stem cell quiescence, promote transient activation, and keep stem cells undifferentiated [31].

### Fgf10 maintains the distal epithelial progenitor population and drives basal cell differentiation in the trachea and main stem bronchi during lung development

**Figure 6:**
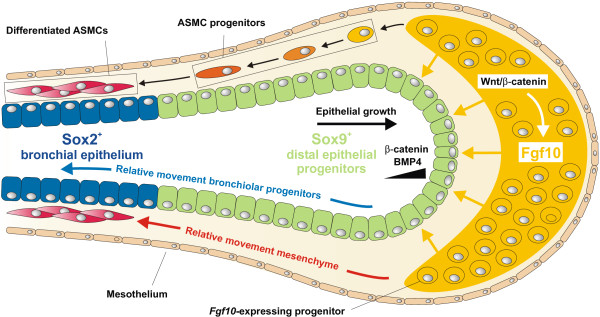
**Fgf10 maintains the distal epithelial progenitor population during early lung development.***Fgf10* is expressed in the distal (submesothelial) mesenchyme of the early developing lung and acts on the distal Sox9^+^ epithelial progenitor cells to maintain them and keep them from acquiring bronchiolar Sox2^+^ fate. Fgf10 directly activates β-catenin signaling in the distal epithelial progenitor cells, and induces *Bmp4* and *Sox9* expression. As the epithelial tube grows towards the source of *Fgf10* expression, progeny from the distal epithelial progenitor cells leave the distal niche-like environment, assuming a more proximal position along the developing airway and start to express the proximal epithelial marker *Sox2*. A subset of *Fgf10*-expressing cells in the distal mesenchyme are progenitors for airway smooth muscle cells (ASMCs) and their amplification, as well as *Fgf10* expression, is dependent on mesenchymal Wnt/β-catenin signaling.

During lung development, a complex interplay between endoderm and mesoderm defines early developmental competence and cell fate. This cross-talk is orchestrated by key signals including Fgfs, Wnts, retinoic acid, Shh and Tgfβ (reviewed in [32]). Proximal-distal patterning of the lung is accompanied by the gradual restricted ability of developmental progenitors to generate the various epithelial lineages in the mature organ [33]. During early lung development, Fibroblast growth factor 10 (*Fgf10*), which is expressed in the distal mesenchyme and is regulated by Wnt signaling [34-36], acts on the distal lung epithelial progenitors to maintain them and prevent them from differentiating into proximal (airway) epithelial cells by inducing *Sox9* and repressing *Sox2* expression [37,38] (Figure 6). Fgf10 is presented as a dimer, bound to heparin sulfate (HS) proteoglycans in the basement membrane (BM), to its receptor Fgfr2b on distal epithelial progenitor cells [39-42]. However, as the lung epithelium grows out, more proximally located cells become further displaced from this distal source of Fgf10 and gradually start to differentiate [33,37,38,42,43]. Precisely regulated *Fgf10* expression and presentation via HS proteoglycans is necessary during early development so that high levels are achieved distally to promote rapid expansion of embryonic progenitors. Cell surface-tethered HS chains play pivotal roles in the local retention of Fgf ligands and can spread Fgf signaling to adjacent cells within a short range [44]. Vice versa, *Fgf10* suppression around the developing airway, as well as during late gestation and postnatal development, is crucial to allow for proper maturation of the lung epithelium [39-41,43-45]. In addition, ectopic *Fgf10* overexpression at later stages of lung development prevents the alveolar differentiation program through the induction of *Sox9* expression [43,46,47]. Interestingly, a subset of *Fgf10*-expressing cells in the distal mesenchyme during early lung development are progenitors for airway smooth muscle cells (ASMCs) [35,48,49] (Figure 6) and lipofibroblasts (LIFs) at later stages [50].

*Fgf10* is also expressed in between tracheal cartilage rings during lung development [51-53]. In the trachea, Fgf10 signaling plays an important role in basal cell differentiation and maintenance during mouse lung development [43]. *Fgf10* knockout tracheas show a 50% reduction in basal cells compared to wild type tracheas [43]. In addition, overexpression of *Fgf10* during lung development results in the ectopic differentiation of a large subset of Sox2^+^ airway epithelial cells into the basal cell lineage all along the conducting airway. Interestingly, inhibition of Fgf signaling from E15.5 until E18.5 resulted in a failure to maintain basal cells in the mouse trachea [43].

### Epithelial stem cell niches in the adult lung

Much progress has been made in identifying the different stem cell populations involved in the maintenance and repair of the adult lung epithelium as well as their lineage relationships. However, much less is known about the nature of stem cell niches in the lung and how they influence stem cell behavior.

Cellular and molecular mechanisms important during development are often reactivated during adult injury repair and in disease. As such, we have recently identified ASMCs as a niche for club stem cells [54]. After naphthalene-mediated airway epithelial injury, surviving epithelial cells secrete Wnt7b in order to activate ASMCs by inducing their c-Myc-mediated proliferation and *Fgf10* expression [54,55], which recapitulates a progenitor-like state (Figure 3A). Fgf10 secreted by the niche then acts reciprocally on surviving club stem cells near NEBs and at the BADJ, imparting stem cell characteristics by inducing a transient epithelial-mesenchymal transition (EMT). As a result, club stem cells break quiescence, induce proliferation and initiate epithelial repair. Short-term *Fgf10* overexpression enhances airway epithelial regeneration, whereas inhibition of Fgf10 signaling reduces regenerative capacity after naphthalene injury [54,56], underscoring the central role of this signaling pathway in regeneration of the bronchiolar epithelium. Interestingly, this re-expression of *Fgf10* by ASMCs is observed after different types of airway epithelial injury, including ozone and bleomycin injury [54].

In the mouse trachea, label-retaining basal stem cells preferentially reside in SMG ducts and in intercartilage regions [57,58], two areas where *Fgf10* expression is the highest [51-53,59]. Whether Fgf10 regulates SMG duct stem cells in the adult lung [11] remains to be investigated.

Interestingly, direct cell-cell contact between basal cells and club stem cells in the tracheal epithelium inhibits the reprogramming of club stem cells into basal stem cells [13]. In this context, basal stem cells themselves contribute to the club cell niche in the trachea. However, it is currently unclear what prevents club cells from reprogramming into basal cells in the lower mouse airways where basal cells are absent.

A novel subpopulation of Scgb1a1^−^ club cells, characterized by expression of N1ICD, Scgb3a2, Uroplakin 3a (Upk3a) and SSEA-1, has been identified juxtaposed to *Ascl1*-expressing NEBs and remain largely uncommitted during development but can give rise to Scgb1a1^+^ club and ciliated cells in the adult lung [60,61]. This cell population is lost in *Ascl1*^*−/−*^mice suggesting that the induction and/or maintenance of these club-like precursor cells is highly dependent on the presence of NEBs in the developing bronchi [60,61]. These findings together with the observation that a naphthalene-resistant subpopulation of club cells is associated with NEBs [16,62] led to the hypothesis that pulmonary NEBs form a stem cell niche essential for club cell regeneration during lung injury. However, lungs lacking NE cells regenerate normally after naphthalene injury [8]. Therefore, further studies are needed to establish whether NEBs indeed provide signals to keep the adjacent naphthalene-resistant club cell population in a more progenitor-like state.

A similar cross-talk between epithelial stem cells and their niche is essential to maintain and regenerate the distal respiratory epithelium [63-65]. In the adult lung parenchyma, LIFs are found juxtaposed to ATII cells and are thought to contribute to the ATII stem cell niche, maintaining their stemness [9]. LIFs assist in the production of pulmonary surfactant by assimilating neutral lipids and transferring triglycerides to ATII cells for final processing of the surfactant [66-71]. In addition, LIFs also express *Fgf10*[50,54], which has been shown to act directly on ATII cells via Fgfr2b to drive the expression of *Sftpc*[43,54,56,72,73]. As such, Fgf10 acts as a protective and therapeutic agent against bleomycin-induced pulmonary fibrosis [56]. Similarly, clonal expansion of the Sftpc^−^ alveolar stem cells enriched for α6/β4 integrins relies on co-culture with Sca1^+^ mesenchymal cells, which can be substituted by exogenous Fgf10 [28]. An additional population of CD166^−^ and Fgf10^+^ adult lung mesenchymal stromal cells (MSC) exists, which functions as a progenitor for LIFs and can support lung epithelial stem cell growth *in vitro* due to its *Fgf10* expression. As during lung development, *Fgf10* expression in these cells can be inhibited by Tgfβ signaling [34,74,75].

Bmp4 secreted by BASCs after bleomycin injury acts on endothelial cells via Bmpr1a to trigger calcineurin/NFATc1-dependent expression of thrombospondin-1 *(Tsp1)*. This endothelial-derived Tsp1 then drives the differentiation of BASCs into the alveolar lineage via a feedback loop [22]. Interestingly, after naphthalene injury, *Bmp4* and *Tsp1* expression is downregulated, which was shown to favor the differentiation of BASCs into the club cell lineage [22]. Considering that *Bmp4* is a main target gene of Fgf10 signaling [73,76-78], it is currently unclear how Fgf10 might be involved in this process since *Fgf10* expression is upregulated in ASMCs and Fgf10 signaling in BASCs is activated after both naphthalene and bleomycin injury [54] (Figure 4C).

Lastly, a recent study provides insight in how the human Lgr6^+^ alveolar epithelial stem cell niche is formed and maintained to effectively ensure stem cell self-renewal capacity [79]. The authors identified a paracrine circuitry in which basal levels of SDF-1 secreted by Lgr6^+^ stem cells recruit and prime fibroblasts to release TNFα. This TNFα then leads to the activation of a TGFβ/p38α-mediated autocrine loop in Lgr6^+^ stem cells, which further enhances SDF-1 production. These high SDF-1 levels then stimulate fibroblasts to produce angiogenic factors to promote angiogenesis [79].

### The role of cell-matrix and cell-cell adhesion in the epithelial stem cell niche

As described above, crosstalk between stem cells and their niche crucially depends on paracrine and autocrine signaling molecules. However, the behavior of epithelial stem cells depends on the integration with several other signals as well, including direct contact with the underlying ECM as well as with neighboring epithelial cells (Figure 5).

Components of the ECM, including fibronectin, proteoglycans and collagens, anchor stem cells in their niche and provides them with mechanical signals, based in part on substrate rigidity [80,81]. This allows stem cells to respond to physical stimuli such as mechanical stress. In addition, the ECM acts as a growth factor reservoir by its ability to bind locally secreted growth factors (for example, Fgf10 and Tgfβ) and other stem cell regulatory molecules (Figure 5), which can be released by proteases such as heparinase [82]. Integrin-mediated cell adhesion to the ECM in the niche guides stem cell fate decisions, including choices between quiescence or proliferation, self-renewal or differentiation, migration or retention, and cell death or survival [83]. For example, loss of contact with the ECM or reduced integrin expression in adherent cells triggers terminal differentiation of cultured epidermal stem cells [84,85]. The pulmonary ECM is subjected to a continuous turnover of more than 10% of the total ECM per day [86]. Thus a dynamic equilibrium between synthesis and degradation of the pulmonary ECM maintains a physiological balance [87]. Alterations in its composition (for example, in response to injury) lead to changes in cell shape and behavior [88], altered binding affinity or cellular distribution of cell-surface receptors [89], and different cellular responses to growth factors [90]. The BM, a specialized form of ECM separating the epithelium from the mesenchyme, is a dynamic structure produced by collaboration between stromal fibroblasts and epithelial cells. Depending on the composition and physical characteristics of the BM, different growth factors can have completely different cellular outcomes, such as cell proliferation, growth arrest, differentiation or apoptosis [91]. Moreover, tissue engineering experiments suggest that a decellularized matrix is capable of dictating exogenous stem cell fate [92], which highlights the importance of integrin-mediated stem cell-ECM communication in controlling stem cell behavior. Bidirectional signaling between epithelial stem cells and the cellular and acellular components of their niche is essential for normal tissue homeostasis, and there is clear evidence that stem cell integrins and BM proteins are involved in this communication [93].

In contrast to our increasingly extensive knowledge of pathways that regulate stem cell properties in the lung during development and regeneration, not much is known about the molecular mechanisms that keep lung stem cells largely quiescent during homeostasis or in check after a regenerative response to prevent tissue overcrowding and dysplasia. Preservation of the quiescent state by the niche is an actively regulated process that maintains the number and function of stem cells [94]. Epithelial cells form polarized cell layers that function as barriers capable of interacting with the underlying matrix as well as with neighboring epithelial cells. Epithelial cell polarity is maintained through the combined action of three major regulatory complexes: Crumbs complex, Par complex and Scribbled complex [95-97]. These complexes are important in organizing cell-cell and cell-matrix adhesion junctions, which have emerged as major signaling platforms that mediate the cross-talk between neighboring epithelial cells and the underlying ECM, thereby regulating epithelial cell differentiation and proliferation via the Hippo pathway [95,96,98-105]. For example, cultured cells arrest proliferation and cell division when the culture becomes confluent. This contact inhibition mechanism is crucial *in vivo* to regulate organ size and its loss is a hallmark of solid tumors [106,107]. An intriguing picture is emerging in which stem cell progeny are a critical niche component, providing essential feedback to their stem cell parents to control stem cell activity and behavior [30]. However, the exact mechanisms underlying (integrin-mediated) mechanotransduction by the ECM and contact inhibition in the lung remain poorly understood.

### Epithelial stem cells and their niches in chronic lung diseases

Chronic lung disorders, such as IPF, asthma and chronic obstructive pulmonary disease (COPD), are characterized by the progressive remodeling of the airways and/or parenchyma, which irreversibly leads to lung dysfunction. In most cases their etiology is not well understood and current therapeutics are aimed at ameliorating rather than curing these diseases. Tissue architecture remodeling involves pathological changes in the composition and physiological function of the epithelium. During normal homeostasis as well as after injury, lung epithelial stem cells need to give rise to the right amount of differentiated progeny while maintaining their self-renewing ability depending on the physiological context. Disrupting this balance can lead to excessive differentiation and stem cell exhaustion, hypoplasia or squamous metaplasia. Such abnormal changes in stem cell function may result from inherent (epi)genetic changes or a dysfunctional stem cell niche. Furthermore, pathological changes to the lung epithelium may drastically influence the crosstalk between stem cells and their niche, which further contributes to disease progression by inducing abnormal changes in mesenchymal components, such as ASMC hyperplasia and fibroblast activation leading to excessive ECM deposition and fibrotic scarring. Chronic injury and prolonged activation of repair pathways are thought to result in decreased repair potential by exhausting the stem cell pool, which leads to defective repair and progressive airway remodeling by promoting a fibrotic response [63].

#### Asthma

Pathological hallmarks in asthma are an increase in smooth muscle mass surrounding the airway wall, SMG hyperplasia and thickening of the basal lamina beneath the seemingly ‘normal’ BM [108]. The latter is accompanied by an increase in the number and activity of subepithelial myofibroblasts with their capacity to lay down new matrix [109,110]. There is also overwhelming evidence for basal cell hyperplasia [111,112] and goblet cell metaplasia (GCM) [113] in the asthmatic conducting airways. In asthmatic patients, goblet cells spread down to the more peripheral airways, where they normally do not exist [114,115]. GCM is not due to the proliferation of pre-existing goblet cells but rather to the transdifferentiation of club cells to goblet cells [116-118]. In fact, goblet cells can actually be regarded as club cells secreting a lot of mucins [119-122].

New paradigms in asthma research point to a central role for the epithelium and chronic activation of epithelial repair pathways as a major cause of airway remodeling and the development of persistent alterations in airway function [123]. This is a shift away from the traditional view in which T-helper 2 (Th2)-type inflammation is considered to be a primary cause in asthma pathogenesis. At its onset, asthma is associated with structural changes in the airways often in the relative absence of airway inflammation [124-126]. A new paradigm for persistent asthma is emerging of a damaged epithelium that repairs incompletely and leads to a chronic wound scenario with the secretion of a range of growth factors capable of driving structural changes linked to airway remodeling [127-129]. Thus, asthma may primarily be an epithelial disorder and its etiology as well as its clinical manifestations could likely be caused by altered epithelial physical and functional barrier properties rather than being purely linked to allergic pathways. In support of this, naphthalene-mediated airway epithelial injury has recently been shown to cause airway hyper-responsiveness [130]. A direct link between a compromised barrier function and allergy has been supported by recent findings in atopic dermatitis. Mutations in the epidermal barrier protein filaggrin (encoded by the *Flg* gene) lead to a defect in epithelial barrier function resulting in increased epithelial permeability and penetration of exogenous substances [131,132]. Disruption of the columnar epithelium by breaking tight junctions or not reforming them efficiently enables tissue damaging agents and infectious particles to penetrate the airway wall, which facilitates toxic, immune, and inflammatory responses accompanying tissue damage [133-135]. There is also a link between a compromised epithelial barrier function and the induction of GCM. Epidermal growth factor (EGF), a key epithelial cell-derived factor that promotes GCM, is normally secreted on the basolateral side of the epithelium and sequestered from its receptor on the apical side through intact adherens and tight junction barriers between neighboring epithelial cells [123,136,137]. The recent conceptual change in asthma pathogenesis raises the interesting possibility that disordered epithelial signaling pathways not only drive susceptibility to environmental insults but also the inflammation and remodeling responses that follow. Similarities between organ morphogenesis and wound healing responses have led to a new concept in which chronic airway inflammation is supported by the structural components of remodeling through activation of the epithelial stem cell-niche unit [138].

Interestingly, the Wnt pathway is induced in ASMCs in a mouse model for asthma [139], whereas epithelial Fgf10, as well as downstream Notch signaling, has been shown to be implicated in mucous/goblet cell hyperplasia [37,119]. In addition, both Fgf10 and downstream Notch signaling are involved in maintaining club stem cells to prevent them from differentiating into terminally differentiated ciliated cells [43,119,140]. These findings suggest that under conditions of chronic injury, the lung may invoke the same signaling pathways which are activated in response to acute injury [54]. Unlike human lungs, mouse lungs only have very few goblet cells in the upper airways. Fgf10 secreted by ASMCs, possibly by activating the Notch pathway, can induce club cell to goblet cell transdifferentiation in the repairing upper airway after naphthalene injury in the mouse [54]. Thus, chronic activation of Wnt-Fgf10 epithelial-mesenchymal crosstalk is likely involved in ASMC proliferation and airway remodeling observed in asthma patients. The sustained stimulation of these pathways may eventually lead to epithelial stem cell depletion and fibrosis. In this regard, it is interesting to note that *Snail1* expression is induced downstream of Fgf10 or Notch signaling in club stem cells during airway epithelial regeneration resulting in a transient EMT [54]. Interestingly, use of a mouse model of acute liver fibrosis on a hepatocyte-specific *Snail1* knockout showed that Snail1 plays a crucial role in the progression of liver fibrosis without driving a full EMT. Instead, Snail1 was shown to drive the expression of growth factors, ECM components and pro-inflammatory mediators [141].

#### Idiopathic pulmonary fibrosis

IPF is a devastating, age-related lung disease with an unknown etiology that is refractory to treatment and has a poor survival rate. Widespread damage to the epithelium and/or exhaustion of the epithelial stem cell pool, such as in IPF patients with certain telomerase mutations [142-149], ultimately leads to epithelial loss. This results in a denuded basal lamina, serum protein exudation and remodeling of the underlying ECM, mediated by fibrotic scarring [63,64,150]. Similar to asthma, IPF was once thought to be driven by chronic inflammation. However, the efficacy of anti-inflammatory or immunosuppressive drugs is inadequate, which urged reassessing the role of inflammation in IPF. Indeed, current evidence indicates that the fibrotic response may primarily be caused by abnormally activated alveolar epithelial (stem) cells [64]. A main hallmark of IPF is an increase in hyperplastic and hypertrophic ATII epithelial stem cells despite widespread epithelial damage and apoptosis. These cells express mediators such as Tgfβ, which induce the formation of myofibroblast foci through the proliferation and transdifferentiation of resident mesenchymal (niche) cells and the attraction of circulating fibrocytes [23,64]. The myofibroblastic foci secrete excessive amounts of ECM, mainly collagens and fibronectin (FN), resulting in fibrotic scarring and progressive remodeling of tissue architecture reminiscent of abnormal repair. Disruption of the tissue-specific epithelial stem cell niche by the fibrotic ECM may further result in aberrant stem cell activation and/or stem cell loss. This prevents proper regeneration and leads to permanent and irreversible tissue scarring, which compromises normal lung function. Abnormal redeployment of developmental epithelial-mesenchymal interactions between epithelial stem cells and their niches have been suggested to play a role in the pathogenic mechanisms that connect IPF with aging and aberrant ATII cell activation [63-65]. After epithelial injury, lung epithelial stem cells and their niches will attempt to restore the damaged epithelium. However, if effective re-epithelialization fails then a process of destructive remodeling and aberrant cellular differentiation is initiated, which produces a dysfunctional disease state shared by several parenchymal pulmonary disorders [63]. IPF patients show a significant loss of ATI cells [64,65]. Moreover, the differentiation of ATII into ATI cells is perturbed in IPF due to aberrant ECM remodeling and changes to the BM structure [64,65]. At this point it is unclear whether there is a correlation between the reduction in ATI cells and a fibrotic response in IPF. However, ATI cells express some important anti-fibrotic factors such as caveolin-1, which is involved in FN turnover [151,152], and mice lacking caveolin-1 or −2 develop spontaneous lung fibrosis [64,151-153].

#### Chronic obstructive pulmonary disease

COPD is another chronic lung disorder that develops mainly in smokers and clinically manifests itself through chronic bronchitis and emphysema. Epithelial remodeling includes basal cell hyperplasia and squamous metaplasia with several layers of Krt14^+^ basal cells, which are rare in the normal epithelium [2]. In addition, mucus hyperplasia is also frequently observed in lungs from COPD patients [2]. Interestingly, *Fgf10* haploinsufficiency is linked to emphysema in COPD in humans, which further emphasizes the significance of *Fgf10* expression in adult lung stem cell niches and its role in homeostasis and regeneration [154].

## Conclusions

Stem cells are indispensible during normal homeostasis and regeneration to restore the form and function of an organ after injury. Great strides are being made toward identifying and characterizing stem cell populations in the adult lung. It is, however, important to keep in mind that stem cells do not act independently to perform these functions. Stem cells are subjected to tight regulatory processes so that they are activated and give rise to the right number and type of differentiated progeny at the appropriate time and place in a given biological context. In that regard, stem cells must be mobilized quickly in response to injury, but once the tissue has been restored, stem cells must also be able to revert back to their quiescent state. How exactly this information is communicated to stem cells is not well understood, but may involve dynamic feedback mechanisms between stem cells and their niche. We are now only beginning to understand the cellular and molecular constituents of the stem cell niches in the lung and how stem cell behavior is influenced by the wide variety of external signals provided by their niche. In this review, we have advocated the involvement of Fgf10, which is expressed in several stem cell niches in the lung, in stem cell maintenance and activation after injury. Developmental pathways are often recapitulated during lung repair and their chronic and sustained activation may lead to lung remodeling observed in chronic diseases such as IPF, COPD and chronic asthma. As such, determining the regulatory pathways involved in stem cell-niche interactions during adult lung homeostasis and repair after injury will pave the way for a better understanding of the molecular mechanisms underlying these devastating disorders for which there are currently few or inefficient treatments. Moreover, effectively recreating the stem cell niche *in vitro* in organoid cultures will be crucial for long-term expansion of lung stem cells and the development of cell replacement therapies. As such, the feasibility of colon stem cell therapy based on transplantation of colon organoids in a damaged mouse colon has recently been demonstrated [155].

## Abbreviations

ASMC: airway smooth muscle cell; ATI: alveolar type I; ATII: alveolar type II; BADJ: bronchioalveolar duct junction; BASC: bronchioalveolar stem cell; BM: basement membrane; COPD: chronic obstructive pulmonary disease; DASC: distal airway stem cell; EGF: epidermal growth factor; ECM: extracellular matrix; EMT: epithelial-mesenchymal transition; FGF10: fibroblast growth factor 10; FN: fibronectin; GCM: goblet cell metaplasia; HS: heparin sulfate; IPF: idiopathic pulmonary fibrosis; Itg: integrin; Krt: cytokeratin; LIF: lipofibroblast; NE: neuroendocrine; NEB: neuroendocrine body; SMG: submucosal gland; Th2: T-helper 2; Trp63 or p63: transformation-related protein 63; Tsp1: thrombospondin-1; Upk3a: uroplakin 3a.

## Competing interests

The authors declare that they have no competing interests.

## Authors’ contributions

TV and SDL developed the review concept, performed the literature review, created figures, wrote and revised the manuscript. Both authors read and approved the final version of the manuscript.

## Authors’ information

TV is a predoctoral candidate. SDL is an associate professor of pediatrics.
